# Interaction of Cholesterol with Perfringolysin O: What Have We Learned from Functional Analysis?

**DOI:** 10.3390/toxins9120381

**Published:** 2017-11-23

**Authors:** Sergey N. Savinov, Alejandro P. Heuck

**Affiliations:** Department of Biochemistry and Molecular Biology, University of Massachusetts, Amherst, MA 01003, USA; ssavinov@umass.edu

**Keywords:** cholesterol-dependent cytolysins, Perfringolysin O, cholesterol, cholesterol-binding

## Abstract

Cholesterol-dependent cytolysins (CDCs) constitute a family of pore-forming toxins secreted by Gram-positive bacteria. These toxins form transmembrane pores by inserting a large β-barrel into cholesterol-containing membranes. Cholesterol is absolutely required for pore-formation. For most CDCs, binding to cholesterol triggers conformational changes that lead to oligomerization and end in pore-formation. Perfringolysin O (PFO), secreted by *Clostridium perfringens*, is the prototype for the CDCs. The molecular mechanisms by which cholesterol regulates the cytolytic activity of the CDCs are not fully understood. In particular, the location of the binding site for cholesterol has remained elusive. We have summarized here the current body of knowledge on the CDCs-cholesterol interaction, with focus on PFO. We have employed sterols in aqueous solution to identify structural elements in the cholesterol molecule that are critical for its interaction with PFO. In the absence of high-resolution structural information, site-directed mutagenesis data combined with binding studies performed with different sterols, and molecular modeling are beginning to shed light on this interaction.

## 1. Introduction

Perfringolysin O (PFO) is the prototypical example of a family of Gram-positive bacterial pore-forming toxins known as the cholesterol-dependent cytolysins (CDCs) [1,2,3]. Despite being present in a broad range of species, most CDCs show an amino acid sequence identity greater than 39% when compared to PFO [2]. The C-terminus (domain 4 or D4) of PFO is responsible for the cholesterol-dependent membrane binding and is the domain with the highest percentage of amino acid identity among CDC members. Cholesterol recognition via D4 is a distinguishing feature of the CDCs. An exception was found for intermedilysin because it uses the human receptor CD59 as a receptor for membrane targeting [4]. However, intermedilysin still requires cholesterol to form pores in membranes [5].

It has long been known that a high level of cholesterol is required in membranes to trigger PFO binding [6,7,8]. More recently it was shown that how much cholesterol is required to trigger binding depends on the overall lipid composition of the membrane [9,10]. However, the precise mechanism by which cholesterol triggers binding and the conformational changes that lead to pore-formation are unknown. In this work we will review our current knowledge on CDC-cholesterol interaction and present some additional insights on the interaction between cholesterol and PFO.

### 1.1. Structural Elements of Domain 4 Involved in Cholesterol Recognition

**Figure 1:**
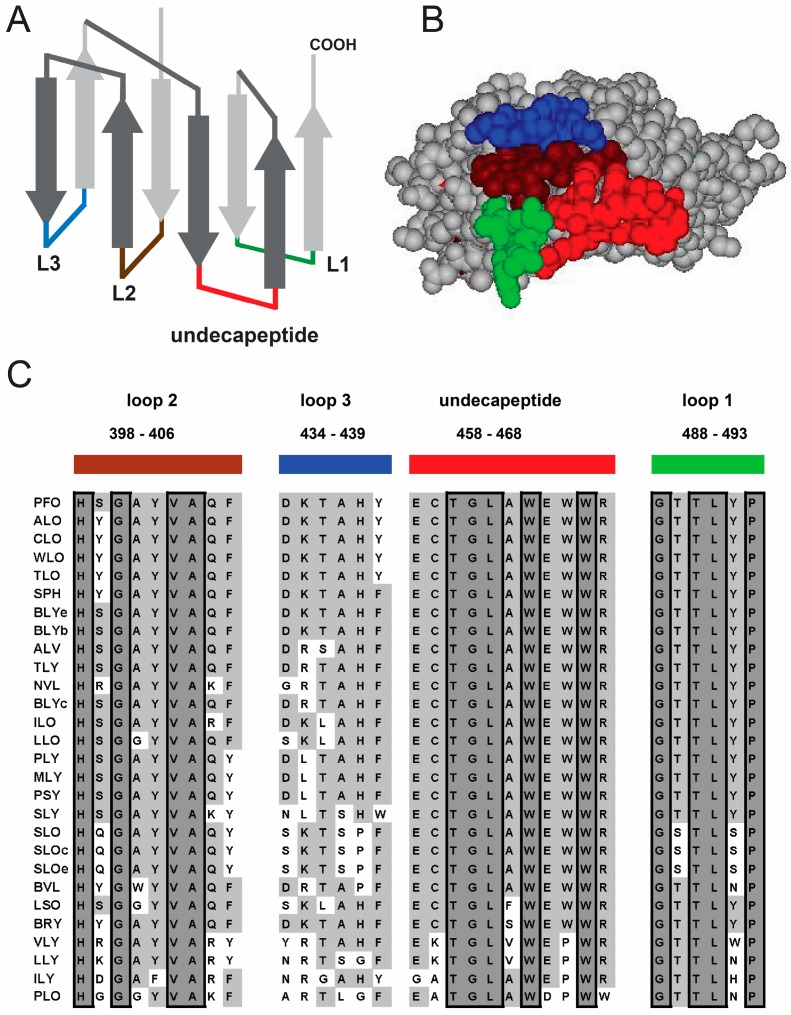
The loops at the tip of D4 are highly conserved among CDCs. (**A**) Cartoon representation of the PFO D4 β-sandwich showing the location of the loops and the conserved undecapeptide. The undecapeptide was colored red and the loops were colored green (L1), brown (L2), and blue (L3); (**B**) A view of the tip of PFO D4 from the bottom showing the loops and undecapeptide color coded as in A; (**C**) Sequence alignment of the 28 CDC family members showing the conserved amino acids boxed and with dark grey background. Highly conserved amino acids are shown with a light grey background. Protein names were abbreviated as defined in [2].

PFO D4 consists of two four-stranded β-sheets located at the C-terminus of the protein (Figure 1A). There are four loops that interconnect the eight β-strands at the distal tip of the toxin. These loops insert into the membrane upon binding and are presumably responsible for the interaction of the toxin with cholesterol [11,12,13]. Two of these loops (L2 and L3) connect β-strands from opposite β-sheets, while L1 and the undecapeptide connect β-strands from the same β-sheet. L1 and the undecapeptide are parallel to each other and abutted perpendicularly by L2, forming a pocket in the bottom of the protein (Figure 1B). The loops that form this pocket are the most conserved segments in D4, and modifications to any of these three loops affect the cholesterol binding properties of PFO [13,14,15,16]. The remaining L3 is less conserved (Figure 1C). Interestingly, a similar loop arrangement has been recently described for the C-edge loops of the eukaryotic protein arrestin [17], a protein that interacts with G protein-coupled receptors blocking G-protein-mediated signaling and directs the receptors for internalization.

The undecapeptide is the longest and most conserved of the four loops. It was originally thought to be exclusively responsible for cholesterol recognition and binding. This idea was supported by several studies showing that modifications in it greatly decreased the pore-forming activity of the protein [14,18,19,20,21,22,23]. However, more recent studies showed that other loops in D4 are also responsible for cholesterol recognition [13]. The undecapeptide has now been suggested to play a role in both the pre-pore to pore transition [12] and the coupling of monomer binding with initiation of the pre-pore assembly [24]. Dowd and colleagues recently showed that modification of a charged amino acid in the undecapeptide (R468) resulted in complete elimination of the pore-forming activity of PFO and had a significant effect on the membrane binding of the toxin [14,24]. Despite the novel functions assigned to the undecapeptide, its role in binding cannot be neglected since many modifications to this segment have been shown to have a significant effect in toxin-membrane interaction [14].

The L3 is located on the far edge of D4, away from a nascent cavity formed by the undecapeptide, L1, and L2 (Figure 1). Modifications introduced into L3 have been shown either to have a negligible effect on cholesterol interaction, or to decrease the amount of cholesterol required for binding [13,16,25]. These results suggest that L3 plays a limited role in cholesterol recognition, and its effect on binding may be related to nonspecific interactions with the membrane that stabilize the bound form of the monomer (e.g., decreasing the k_off_).

A suggested cholesterol recognition motif composed by only two adjacent amino acids in L1, (T490 and L491 in PFO, Figure 1C) [13], is conserved throughout all reported CDCs. Modifications to these two amino acids greatly affect the binding of the protein to both cell and model membranes [13,26]. These data suggest a prominent role for T490 and L491 in cholesterol recognition, however, other well conserved amino acids located in proximity of the pocket formed by L1, L2, and the undecapeptide have not been analyzed yet (e.g., H398, Y402, A404, E458, and P493) and may also contribute to cholesterol binding.

### 1.2. The Effects of Membrane Lipids on the Cholesterol Threshold Required for CDC Binding

Cholesterol concentrations of more than 30 mol % are usually required to trigger binding of PFO to liposomes prepared exclusively with phosphatidylcholine [8,27]. Other CDCs showed similar effects, for example streptolysin O (SLO) [7], lysteriolysin O [28], and tetanolysin [6]. How much cholesterol is required to trigger PFO binding (or “cholesterol threshold”) is reduced by the incorporation of double bonds in the acyl chains of the phospholipids or by replacing phosphatidylcholine by phospholipids with smaller head groups [9,10,15]. The high level of cholesterol required to trigger PFO binding, the discovery of cholesterol-rich domains in membranes, and the presence of PFO on detergent resistant membranes [29] led some researchers to associate PFO binding with the presence of membrane rafts [30]. However, it is difficult to envision a scenario where cholesterol will be more readily available to interact with PFO if located in a cholesterol-rich domain where the interaction with other lipids is stronger. For example, it has been shown that the presence of sphingomyelin (a lipid that interacts with cholesterol) actually interferes with PFO binding [10]. Recent studies on PFO-cholesterol interaction suggest that accessibility of cholesterol to the membrane surface is the key factor to trigger PFO binding [10,15,31,32]. 

Moreover, despite the influence phospholipids have on the cholesterol-dependent binding of PFO, their presence is not required since cholesterol alone (in the absence of any other lipid) is sufficient to trigger PFO oligomerization and formation of ring-like complexes ([33] and references therein).

### 1.3. Structure Elements of Cholesterol that Influence CDC Activity

**Figure 2:**
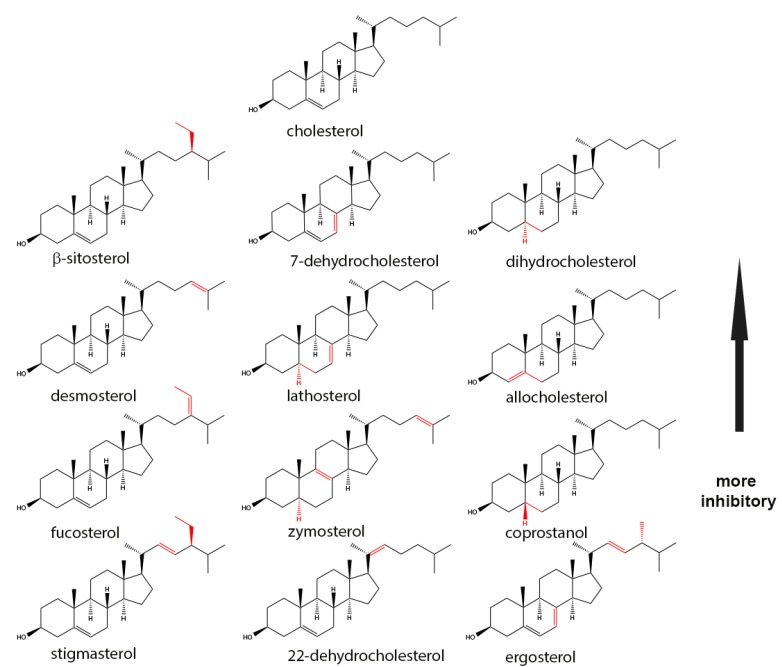
Chemical structure of the sterols that interacts with the CDCs. The differences from cholesterol are highlighted in red. Top molecules inhibit/interact strongly, and the ones in the bottom more weakly (based on data presented on Table 1).

Early studies of the inhibition of SLO and PFO hemolytic activity by different sterols revealed elements of the cholesterol molecule that are critical for its interaction with the CDCs [34,35,36]. The affinity of the toxin for a particular sterol was indirectly estimated by measuring the hemolytic activity of the toxin after a pre-incubation with the sterol. It was assumed that the higher the inhibition, the stronger the affinity for the sterol (Table 1, Figure 2). Results from these studies have been reviewed by Alouf [37] and are briefly summarized below.

#### 1.3.1. The Presence of a Lateral Aliphatic Side Chain of Suitable Size at Carbon 17 Is Required

**Figure 3:**
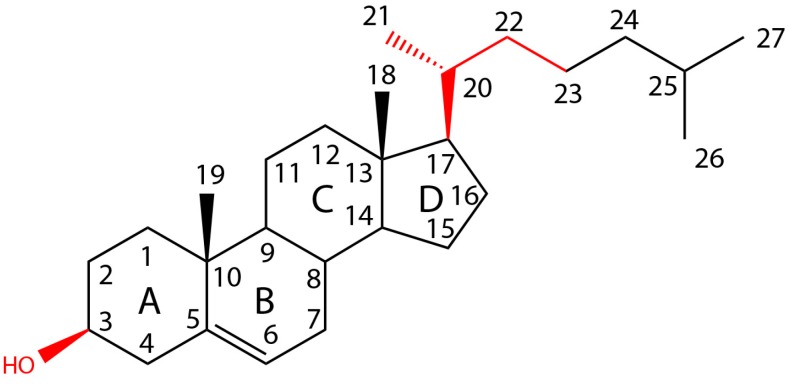
Chemical structure of cholesterol showing the individual rings (**A**)–(**D**) and numbered carbon atoms. Elements identified as critical for the interaction with CDCs are indicated in red.

Addition of polar hydroxyl groups at position C25, C26, or C20 of the eight-carbon chain removes the inhibitory effect (Figure 3). Replacement of the eight carbon acyl chain for a keto group or an acetyl group removes the inhibitory effect. Sterols with a double bond at C24–C25 (desmosterol) or with a =CH-CH3 group at C24 (fucosterol) are still inhibitory. Modification of the eight-carbon chain by introduction of an ethyl group at C-24 (β-sitosterol) is not critical, but the simultaneous addition of a double bond at C22–C23 and either an ethyl group (stigmasterol) or a methyl group (ergosterol) at C24 weakens the inhibitory effect (see Figure 2).

#### 1.3.2. The Presence of a 3 β-Hydroxy Group on Ring A Is Required

The inhibitory capacity of the sterol is removed when the hydroxyl group is eliminated (cholestane), oxidized (cholestanone), esterified (cholesterol acetate), etherified (cholesterol methyl ether), or epimerized into alpha position (epicholesterol). Substitution of the hydroxyl group for a thiol group (thiocholesterol) or chloride (3 chlorocholestene) also removes the inhibitory effect.

#### 1.3.3. An Intact Ring B Is Required

The presence of the A ring with the β-hydroxyl group and the aliphatic chain at carbon 17 are not sufficient for binding if the B ring is open (cholecalciferol). However, neither the saturated or unsaturated state of ring B and the position of double bonds (lathosterol, allocholesterol, or zymostenol) nor the stereochemical relationships of rings A and B to each other are critical for inhibition. The 5β-cis (coprostanol) and 5α-trans (dihydrocholesterol) configurations are both inhibitory.

Similar effects were observed for the inhibitory effect of sterols on SLO and PFO (the inhibition of 7-dehydrocholesterol was higher for SLO, but the sample used in this study presented 3 spots on a thin layer chromatography plate, therefore we need to be cautious when considering this result). An exception was coprostanol, which was a better inhibitor for SLO than for PFO. Interestingly, some amino acids in L1 and L2 differ between SLO and PFO (Figure 1C), suggesting that these loops may interact with the B ring of cholesterol.

Some differences were observed when the sterols were incorporated into model membranes (Table 1) [9], but in this case one also need to consider the differential interaction that each sterol may have with the phospholipids. Oligomerization of PFO on liposomes containing ergosterol or 7-dehydrocholesterol was similar to the one observed with cholesterol, but these sterols quenched the Trp emission [38] and therefore the binding of PFO to liposomes could not be assessed using Trp fluorescence.

In the present work, we explored the interaction of free sterols in solution with PFO using the Trp emission increase that follows D4-sterol interaction [33]. In addition, we study the effect of sterol-binding in the conformational changes that occur in D3. Finally, molecular modeling was attempted to offer structural rationale for the observed trends.

## 2. Results

### 2.1. Selective Solubilization of Sterol Aggregates by Methyl-β-cyclodextrin

**Figure 4:**
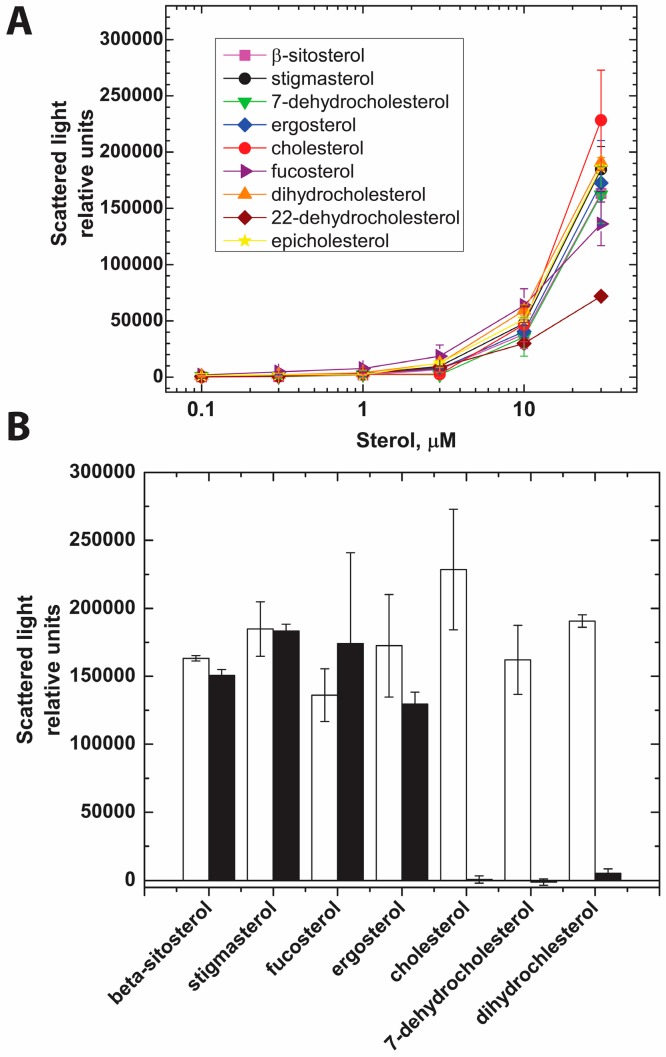
Sterol precipitated when added into aqueous buffer solution and they were differentially solubilized by mCD. (**A**) Scattered light at 500 nm of aqueous buffer solutions containing the indicated amount of sterols. Sterols were added from ethanolic solutions incrementally and incubated 5 min at 37 °C before each measurement; (**B**) mCD (final concentration 3 mM) was added into solutions containing 30 µM sterols and the right angle light scatter measured after 5 min incubation at 37 °C. The bars represent the average of at least two measurements and the error bars correspond to the range. White bars and black bars represent the scattered light before and after incubation with mCD, respectively.

We have shown that PFO is able to bind to cholesterol aggregates in solution. Cholesterol aggregates remain soluble in neutral aqueous buffers, but start to precipitate when the cholesterol concentration reaches the solubility limit (around 4.7 μM) [33,39]. Similar aggregation profiles were observed when other sterols were added into aqueous buffer up to a concentration of 30 μM (Figure 4A) with the exception of 22-dehydrocholesterol, where scatter was lower than that observed for the other sterols used in this study. It is well known that cholesterol interacts with methyl-β-cyclodextrin (mCD), and the addition of mCD solubilizes cholesterol aggregates and microcrystals (Figure 4B) [33]. While attempting to repeat the mCD solubilization with other sterols, we noticed that the sole addition of one or two carbons to C24 in the aliphatic chain of the sterols was sufficient to interfere with this process. No solubilization was observed for aggregates formed by β-sitosterol, fucosterol, stigmasterol, or ergosterol (Figure 2 and Figure 4B), but complete solubilization was observed for cholesterol, 7-dehydrocholesterol, and dihydrocholesterol. The scattered light (relative units) for aggregates formed by 30 μM aqueous solutions of two other sterols lacking modifications to the acyl chain -22-dehydrocholesterol and epicholesterol- was 71,800 and 186,400, respectively. The scattered light for both aggregates decreased more than 96% after addition of mCD, in good agreement with the need for a C20–C25 linear aliphatic chain in the sterol molecule for fast mCD solubilization of sterol aggregates. Yet, it was reported that mCD can bind some C24 substituted sterols if they are added directly into the solution containing mCD [40].

The molecular bases for the sterol-mCD interactions are not well understood, but it is clear from the observations described above that if sterols with group additions to C24 are allowed to form aggregates, they do not interact with mCD in the same way that they do when directly diluted into a solution containing mCD. These results suggest that the order of the addition of the sterols and the protein may influence the outcome obtained for protein-sterol interactions. Therefore, we reasoned that when studying the interaction of sterols with water soluble molecules (like PFO in these studies) it would be necessary to add the sterols into a solution containing PFO to minimize the formation of sterol aggregates, and maximize the exposure of PFO to solubilized sterol monomers.

### 2.2. PFO Interaction with Free Sterols

Liposomes made with different sterols have also been used to study how modifications to the cholesterol molecule affect its interaction with PFO. However, in these studies the PFO-sterol interaction will be influenced by both, the direct interaction (affinity) of the sterol molecule with PFO, and the interaction of the sterol with other membrane components (phospholipids, sphingomyelin, etc.). The higher the interaction of the sterol with other lipids, the less available it will be to interact with PFO. Therefore, to determine what elements of the cholesterol molecule are critical to bind PFO, it is important to perform these studies in the absence of other lipid components. We have shown that binding of PFO to cholesterol in aqueous solution produces an increase in Trp emission, similar to the one observed when the toxin binds to membranes containing cholesterol [33]. We reasoned that the same emission change could be used to analyze the interaction with other sterols.

**Figure 5:**
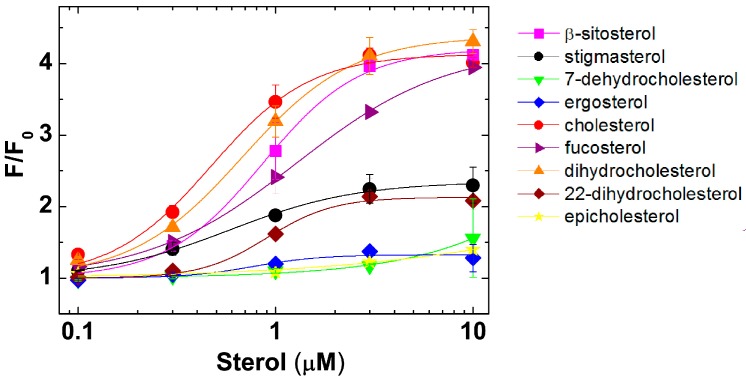
Binding of sterols to the PFO derivative containing the native undecapeptide (nPFO) [33]. Trp emission intensity for 0.1 µM nPFO was measured before (F_0_) and after (F) addition of the indicated amount of sterol. Each data point shows the average of at least two measurements and their range. The cholesterol concentration that produced half of the total Trp emission increase for nPFO was 0.5 μM.

The interaction of PFO with sterols was studied following the Trp emission increase that follows PFO-sterol interaction (Figure 5). No emission increase was observed when PFO was incubated with non-interacting sterols like epicholesterol (Figure 5) [33]. In this analysis, both dihydrocholesterol (reduction of the C5–C6 double bond) and β-sitosterol (addition of an iso-propyl group at C24) showed a concentration-dependent Trp emission profile that was slightly shifted to higher sterol concentration when compared to the one obtained for cholesterol (Figure 5). Similarly, the concentration-dependent change when adding fucosterol was shifted to higher sterol concentrations, indicating that the rigidity introduced by the double bond between C24 and the ethyl group restrict the interaction of the sterol acyl tail with PFO (see Figure 2). These four sterols showed a similar maximal increase in Trp emission when added to a final concentration of 10 μM. A lower Trp emission increase was observed when PFO was incubated with stigmasterol or 22-dehydrocholesterol, suggesting that flexibility between C20–C22 is important for the interaction of PFO with cholesterol. Surprisingly, no Trp emission increase was observed for 7-dehydrocholesterol and ergosterol, two sterols that are able to inhibit the hemolytic activity of PFO (Table 1) [36]. Both of these sterols possess two conjugated double bonds in the B ring. It has been suggested that this double bond quenches the Trp emission eliminating the increase produced upon the interaction of PFO with the sterol molecules [41]. Inner filter effect could also contribute to mask the Trp emission increase because of the overlap between the Trp and sterol absorption wavelengths (Figure S1). Therefore, a different approach was required to analyze the interaction of these sterols with PFO.

**Figure 6:**
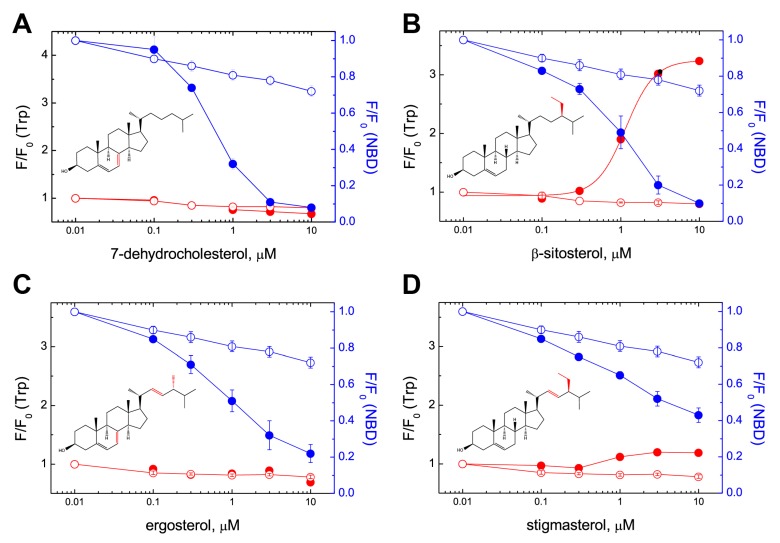
Sterol binding to D4 paralleled the conformational change in D3. rPFO^V322C-NBD^ (0.1 μM) was titrated with (**A**) 7-dehydrocholesterol, (**B**) β-sitosterol, (**C**) ergosterol, (**D**) stigmasterol, and the Trp emission intensity and NBD emission intensity were determined in the same sample at each sterol concentration. Open symbols correspond to a parallel experiment where an identical volume of ethanol was added (no-sterol control). Trp emission data are shown in red, whereas NBD emission data are shown in blue. The average of at least two independent measurements and their range are shown. As a reference, the cholesterol concentration that produced half of the total NBD emission decrease for rPFO^V322C-NBD^ was 0.8 μM [33].

The increase in Trp emission that results from the interaction of PFO D4 with cholesterol is followed by the movement of a short β-strand (β5) in D3 that exposes the monomer-monomer interface required for oligomer formation [42]. This conformational change can be detected using the rPFO^V322C-NBD^ derivative. The environment-sensitive NBD fluorescent probe has a high lifetime (~8 ns) in the monomeric toxin, and NBD lifetime drops to ~1 ns when the protein interacts with cholesterol or cholesterol containing membranes [33]. A decrease in the fluorescence intensity of the NBD dye would be indicative of the interaction of PFO with sterols even if the spectroscopic properties of the sterol molecule interfere with the increase of Trp emission (as it is the case for 7-dehydrocholesterol and ergosterol). Using this assay, we observed that both 7-dehydrocholesterol (extra double bond in ring B) and ergosterol (the same extra double bond in ring B plus another double bond at C22 and a methyl group at C24) triggered the NBD emission decrease when incubated with the rPFO^V322C-NBD^ derivative (Figure 6A,C). We also tested β-sitosterol and stigmasterol, two sterols that showed a strong and weak interaction with PFO, respectively (Figure 6B,D). In both cases the decrease in NBD emission was parallel to the increase of Trp emission. These data indicate that the conformational change in D3 is also a good reporter for the interaction of PFO with different sterols.

### 2.3. Molecular Modeling Rationales for the Observed Cholesterol Structure–Activity Relationship and Mutagenesis Data

To rationalize the data from both the site-directed mutagenesis studies and Structure–Activity Relationship (SAR) analysis of sterols, we have undertaken a modeling study using available CDC structures as receptors and cholesterol as a ligand. The realization that conformational changes need to occur to create an arrangement capable of association with cholesterol, prompted us to employ an Induced Fitting Docking (IFD) algorithm [43] (Schrödinger, LLC, New York, NY, USA). The IFD algorithm iterates docking stages with local minimizations to identify a likely binding site on the surface of D4. We have selected the following criteria for judging the likelihood of putative binding arrangements: (i) contact with highly conserved residues of D4 (due to mechanistic similarities in cholesterol effects among CDCs); (ii) engagement of both the conserved Thr-Leu pair on L1 and undecapeptide (as determined through site-directed mutagenesis); and (iii) participation of equatorial hydroxyl, the only polar site in cholesterol, in H-bonding interaction with conserved donors, acceptors, or both.

Our initial attempts with the PFO structures available from Protein Data Bank (PDB, ID: 1PFO, 1M3I, and 1M3J) failed to yield any reasonable binding arrangements that would satisfy the criteria stated above. Further analysis of these and other structures of CDCs highlighted the unique conformation that the undecapeptide adopts in PFO [26,44,45], which is curled up against the exposed face of the β-sandwich. The undecapeptide is kept in this conformation by an edge-to-face stacking of conserved W464 with Y432, a residue that is unique to PFO among the various solved CDCs x-ray structures. This arrangement separates the undecapeptide from L1 with its Thr-Leu pair, critical for cholesterol association [13], and was, therefore, not as suitable as a starting point for these studies without undergoing a major conformational reorganization.

**Figure 7:**
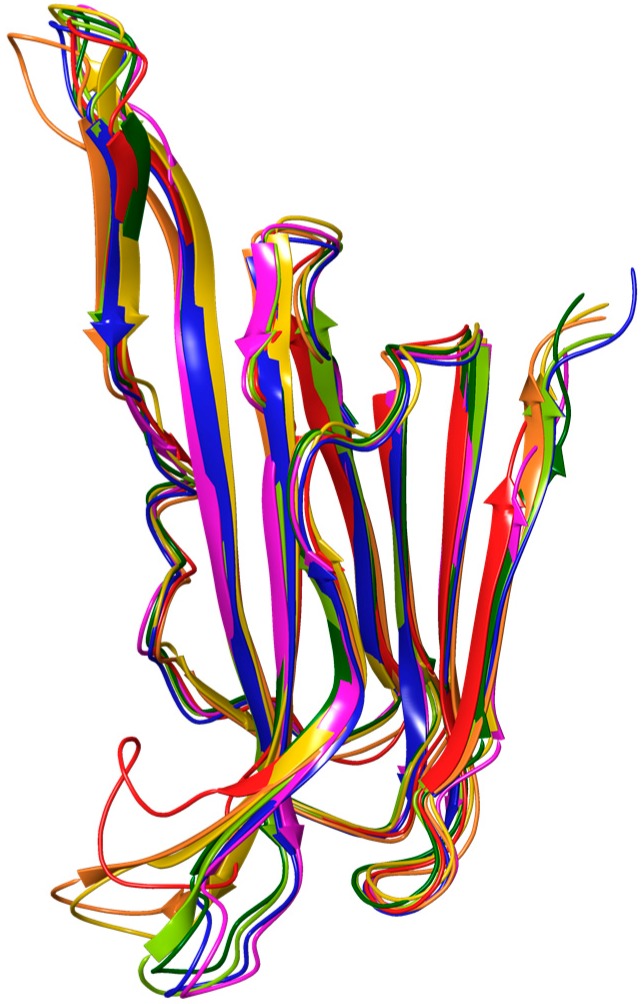
Overlay of PFO (red) and PLY (orange through violet) structures from the PDB with variable level of loop unravelling by the conserved undecapeptide motif (1PFO: red, 5CR8 chain D: orange, 5CR8 chain A: yellow, 5CR6: yellow-green, 5AOD: green, 5AOF: blue, and 4ZGH: violet). The PLY structure with the most proximal disposition of undecapeptide and L1 loop (4ZGH) was used as a starting point for flexible docking of cholesterol.

As a consequence, we have expanded the search to suitable structural models for docking studies to all CDCs featuring the intact undecapeptide sequence from PFO. This search yielded eleven X-ray crystal structures from the PDB with resolutions of at least 3.1 Å: anthrolysin O (1 structure), listeriolysin O (1), SLO (1), suilysin (1), and pneumolysin (PLY) (7). Unlike the PFO structures, the seven structures of PLY offered a rather wide diversity of conformational solutions for the undecapeptide (Figure 7). PLY has a high level of conservation with PFO in the membrane-associating regions (91% identity, 97% similarity for the loops in D4, Figure S2). The conformations of the undecapeptide in PLY ranged from a nearly canonical β-hairpin with _430_GLAW_433_ reverse turn projected away from L1 (PDB ID: 5CR8) to a significantly more unstructured and relatively unraveled loop with multiple solvent-exposed peptide bonds (PDB ID: 4ZGH) that increases the density of hydrophobic residues co-projected toward the membrane (Figure 7). This flexibility is not unexpected for a sequence that contains amino acids uncommon in turns (Leu, Ala, Trp) and strands (Glu, Cys, Gly) [46]. Gratifyingly, in the latter structure, a largely contiguous hydrophobic pocket at the interface of the L1 and undecapeptide is starting to emerge furnished exclusively with conserved residues, several of which come from the rearranged undecapeptide.

**Figure 8:**
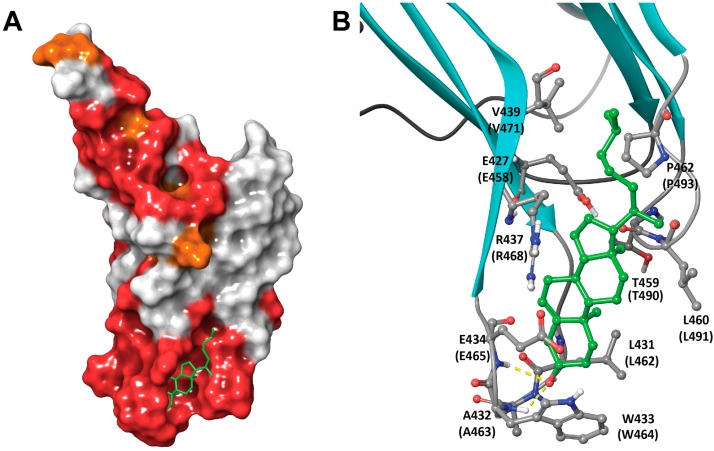
The IFD-proposed model for the cholesterol–PLY-D4 complex. (**A**) PLY D4 is shown in its full size and rendered as molecular surface colored by sequence conservation between PFO and PLY (red: identical, orange: similar, white: non-conserved). The molecule of bound cholesterol is shown as green tubes; (**B**) Close-up of the binding pose predicted by the IFD docking. The binding site of PLY D4 is rendered as ribbons shown as cyan arrows and grey tubes for β-strands and loops, respectively. The key contact residues are shown and labeled with sequence positions for PLY and PFO (in parenthesis). The cholesterol is shown as a green ball-and-stick model, and H-bonds between the hydroxyl of cholesterol and undecapeptide backbone are shown as yellow dashed lines.

Upon flexible IFD, the model derived from 4ZGH has yielded a set of related binding poses that were compatible with binding arrangement criteria, cholesterol SAR, and mutagenesis data. Contrary to the previously published models [14,26], the binding pose predicts that cholesterol undergoes a flip from the membrane arrangement (Figure 8A), which places the equatorial hydroxyl of cholesterol within the undecapeptide in multiple H-bonding contacts with the backbone amides (Figure 8B), providing an explanation for the rigorous H-bonding-enable hydroxyl requirement in cholesterol variants capable of hemolysis inhibition (Table 1). This flip could be coupled to the overall inward movement of the undecapeptide loop as it approaches L1 from the curled away positions. In the model, the B-ring plays a role of a rigid spacer between the A ring and C & D rings engaging the conserved L1 residues. Hence, while its rigid cyclic nature is critical for the display of important recognition elements., (e.g., cholecalciferol) [34], the saturation status and even bridgehead stereochemistry are not expected to have a significant effect on binding, as seen with lathosterol, allocholesterol, zymostenol, coprostanol and dihydrocholesterol. The remainder of the polycyclic core of the sterol (rings C and D, Figure 3) is found in contact with the conserved Thr-Leu pair (T490-L491 in PFO or T459-L460 in PLY) in L1, proven essential for binding of cholesterol in previous studies [13]. The aliphatic tail, in turn, interdigitates with similarly aliphatic and conserved V439 and P462 in PLY (V470 and P493 in PFO). The extended binding pose adopted by cholesterol explains the need for the equatorially projected hydroxyl, which is capable of making multiple H-bonding contacts, unlike a largely occluded axial hydroxyl in epicholesterol [33]. Two other notable events occur during the IFD search to permit the association: (i) the terminal residue of the undecapeptide (R437 in PLY and R468 in PFO), a residue critical for the stability of the toxin structure [47], becomes solvent exposed and opens up the space between two β-sheets to become available for association with cholesterol; and (ii) the first residue of the undecapeptide, (E427 in PLY and E458 in PFO), having lost its salt-bridge partner while remaining in a largely hydrophobic environment is expected to become protonated with a concomitant pKa shift from ~4.5 in PFO or β-hairpin PLY structures to 6.5 and 7.5 in cholesterol-free and bound 4ZGH-derived models, respectively (Epik, Schrödinger, LCC, New York, NY, USA) [48], making the binding surface even more hydrophobic and therefore, more welcoming to a ligand as lipophilic as cholesterol. This observation is consistent with the report of low pH (5.5–6) enhancing PFO–membrane association [9]. This model provides a valuable set of testable hypotheses for further evolution of our insight into mechanistically complex events occurring prior to pore formation by CDCs.

## 3. Discussion

Despite the various X-ray high resolution structures available for CDCs, the structure of the toxin bound to cholesterol has remained elusive. The only information available about the interaction of CDCs with cholesterol has been obtained by combining effects of site directed mutagenesis and structurally distinct sterols. Site directed mutagenesis provided information about how different amino acids contribute to cholesterol binding (assuming that amino acid substitutions do not significantly affect the folding of the protein). The use of different sterols showed how critical different groups or parts of the molecule are for protein interaction. In this work we reviewed the information collected on CDC-sterol interactions and provided some further information about the interaction of free sterols with PFO, a prototypical example of the CDCs. By integrating all the available data, we constructed a binding model for cholesterol–D4 complex that satisfies both the previous findings and those reported herein, and rationalizes the critical need for cholesterol in the membrane-anchoring mechanism of CDCs.

Cholesterol and related sterols (Figure 2) are hydrophobic molecules with very low solubility in water. These sterols precipitate when their concentration increases above 5 μM (Figure 4A). Cyclic sugars, like mCD have been often used to solubilize and transport cholesterol into/from membranes [40]. While most aggregated sterols were readily solubilized by mCD, those with bulky substitutions into the acyl-tail were not (Figure 4B). It is not clear if sterols with additions to their acyl chain are not able to interact with mCD (steric effect) or if the formed sterol aggregates are kinetically trapped in a meta-stable state. Successful binding of mCD to some of the C24 substituted sterols suggest that the latter may be the case [40]. More research is required in this area to elucidate the molecular details for the interaction of sterols with mCD. However, to minimize the potential problems of working with aggregated sterols in these studies, each sterol was added in small aliquots into a solution containing monomeric PFO.

Interaction of PFO with sterols was determined by the increase in Trp emission (Figure 5). Cholesterol, dihydrocholesterol, and β-sitosterol showed a similar profile, which was in agreement with the results obtained using inhibition of hemolysis (Table 1). Stigmasterol, a poor inhibitor of hemolysis, showed a small change in the Trp emission. This weak interaction was corroborated by the small decrease on NBD emission in D3, a conformational change that follows binding (Figure 6D). A similar low interaction profile was obtained for 22-dehydrocholesterol, suggesting that free movement around C22 is required to stabilize cholesterol in its binding site. The limited NBD emission decrease observed for ergosterol suggested an intermediate binding affinity for PFO D4 (similar to that of fucosterol, as determined using Trp emission). Flexibility of the acyl chain seems to be necessary to accommodate the cholesterol molecule into the D4 binding site. NBD emission decrease observed for 7-dehydrocholesterol and cholesterol confirmed the similar interaction observed using Trp emission. In summary, two regions of cholesterol appeared to be critical for the interaction with PFO, the β-hydroxyl group and the flexibility of the acyl chain around C20–C22 (Figure 3).

The extent of the decrease in NBD emission observed for 7-dehydrocholesterol, β-sitosterol, ergosterol, stigmasterol, and the non-interacting epicholesterol correlated with the inhibitory properties of the same sterols when tested using hemolytic activity (Table 1) [33,36]. However, it is possible that hemolysis inhibition is caused by the irreversible oligomerization of the toxin on sterol aggregates (as shown previously for cholesterol) [33], and not by the competition between the sterol and cholesterol for the binding site. The correlation between hemolysis inhibition and cholesterol binding in aqueous buffer is more apparent than the one observed with liposomes. As mentioned above, the interaction of the sterols with lipids may complicate the interpretation of sterol effect on toxin binding when using lipid bilayers.

Binding of a water-soluble PFO monomer to the membrane is diffusional and electrostatic interactions may play a role since it has been observed that the introduction or elimination of charged residues alters binding [12,16,24,25,49]. While on the membrane surface, insertion of non-polar and aromatic amino acids, and/or specific interactions with membrane lipids, help to anchor the protein to the membrane [50]. However, hydrophobic amino acids are rarely exposed on surfaces of water-soluble proteins, and therefore conformational changes are required to facilitate the interaction of these residues with the hydrophobic core of the membrane bilayer. These conformational changes may contribute to generate a non-polar cavity required to fit a cholesterol molecule. Cholesterol has been found located into non-polar protein cavities, for example for the lysosomal protein NPC2, responsible for Niemann-Pick type C disease [51]. No such cavity is apparent in the structure of the water-soluble monomer of PFO. 

The binding model, which was produced via a systematic survey of conformational states of undecapeptide (offered by solved crystal structures of related toxins, Figure 7) and flexible docking, provided a basis for structure-guided rationalization of the cholesterol SAR trends reported herein. Thus, the conserved Thr-Leu pair from L1, essential for recognition of cholesterol, is engaged in the model by the bound ligand, while residues within the undecapeptide, interact with cholesterol via H-bonding contacts through its backbone (Figure 8). The aliphatic–aliphatic contact predicted by the model to be established between cholesterols’ tail and conserved residues at the junction of two β-sheets may accounts for double bond intolerance in the tail, which is expected to reduce conformational flexibility and interfere with the compact interdigitation that the saturated variant is capable of.

Both the pH effect in association and the critical role for the terminal Arg in the undecapeptide can be rationalized by the decoupling of the E458/R468 ionic pair (in PFO, see Figure 8) upon cholesterol recognition. This leads to change in glutamate’s pKa that promotes protonation and reduction in surface polarity of the cholesterol-binding site. The exposed Arg is, in turn, likely important for electrostatic contact with the anionic head-groups of membrane lipids.

This binding model also provides the basis for some of the cholesterol-dependent effects observed when residues in the D4 loops are mutated. For example, T490 in the PFO-cholesterol model is predicted to be involved in a complex H-bonding network involving both its side chain and backbone carbonyl and the side chains of T460 and E458 (in its protonated form) from the undecapeptide. The static model, however, does not provide a simple explanation for the adjacent L491S mutation that does not significantly change the cholesterol-binding of PFO [25]. This implies a newly found H-bonding role for the Ser side chain at this position that, in contrast with the reduced affinity observed for the L491A substitution, conserved the affinity of PFO for cholesterol.

Finally, the cholesterol-assisted quenching of H-bonding capacity of the flexible undecapeptide may have profound outcome on passive diffusibility of this peptide for anchoring at the membrane. Attainment of conformations that allow quenching of H-bonding capacity by peptide bond NH groups has been noted as a critical pre-requisite for passive internalization of cyclic peptides into biological membranes [52]. The energetic basis for this requirement is found in high energy of desolvation of fully exposed peptide bonds upon passage from high-dielectric water to the low-dielectric interior of a membrane that is typically associated with poor membrane permeation of unstructured peptides in general. Hence, the conformational adaptation of the undecapeptide upon association with cholesterol predicted by the model accomplishes several transformations that combine to promote anchoring at the membrane: (i) higher density of hydrophobic residues projected toward the membrane; (ii) enhanced charge complementarity in the form of exposed R468; and (iii) reduced desolvation costs for the internalization of undecapeptide via H-bonding quench with the cholesterol’s hydroxyl. While this model would require further experimental validation, it offers new insights into the D4–cholesterol interaction than can be capitalized in future studies.

## 4. Materials and Methods

### 4.1. Materials

Phospholipids were from Avanti Polar Lipids); β-methyl-cyclodextrin (mCD) from Sigma C-4555 (mean degree of substitution 10.5–14.7), 7-dehydrocholesterol higher than 98% by HPLC Fluka, ergosterol 98% by HPLC Fluka, stigmasterol ~95% GC Fluka, β-sitosterol higher than 98%, and cholesterol from Steraloids.

Preparation of nPFO and rPFO^V322C-NBD^ derivatives was done as described previously [33]. rPFO refers to the use of the Cys less PFO^C459A^ derivative.

### 4.2. Incubation with Sterol Dispersions in Aqueous Solutions

Water-soluble PFO samples (0.3 mL final volume, 0.1 μM final concentration) in buffer C (50 mM Hepes (pH 7.5), 100 mM NaCl, 0.5 mM EDTA, 1 mM DTT) were equilibrated at 37 °C for 5 min before the net initial emission intensity (F_0_) was determined (i.e., after blank subtraction). Sterols were then added to the indicated final concentration, and the sample was then incubated at 37 °C for 15 min. The net emission intensity (F) of the sample was determined after blank subtraction and dilution correction. Sterols were dissolved in absolute ethanol to 10 mM and diluted with additional ethanol as necessary. When added to solutions of nPFO or rPFO derivatives, the final concentration of ethanol was always lower than 5% (*v*/*v*). Control samples were incubated with an identical volume of ethanol. When indicated, sterol aggregates were dissolved by the addition of mCD to a final concentration of 3 mM. mCD was prepared dissolving 83 mg into 1 mL of HBS buffer (Hepes 50 mM pH 7.5, NaCl 100 mM) at 37 °C for 15 min, centrifuged full speed in microfuge and filtered using a 0.22 μm Millipore filter. The solution was stored at 4 °C for no more than a month.

### 4.3. Steady-State Fluorescence Spectroscopy

Intensity measurements were performed using the same instrumentation described earlier [25,33,53]. The excitation wavelength and bandpass, and the emission wavelength and bandpass, were, respectively: 470, 4, 530, and 4 nm for NBD; 295, 2, 348, and 4 nm for Trp; 470; and 500, 1, and 500, 1 nm for right angle light scattering measurements.

### 4.4. Molecular Modeling

All computational procedures were carried out using Schrödinger’s Small-Molecule Drug Discovery suite of programs (v. 2016-1, Schrodinger, LLC, New York, NY, USA): Maestro, Protein Preparation Wizard, Epik, Glide, Prime and Induced Fit. The energy optimized all-atom models were generated via a protonation state assignment (Epik), missing atom/loop reconstitution (Prime, OPLS3 force field) and constrained minimization (Prime) sequence within Maestro’s Protein Preparation Wizard. The flexible docking was initiated by placing the entire loop-region of D4 into a 15 × 15 × 15 Å^3^ grid box. Residues within 5 Å of ligand poses obtained with side-chain-free models (Glide) were refined (Prime) through docking-minimization iterations (Induced Fit).

## Figures and Tables

**Table 1:** Interaction of different sterols with CDCs.

	Hemolysis Inhibition		Sterol in Aqueous Buffer		Sterol in Liposomes	
	SLO	PFO	PFO	PFO	PFO	PFO
			Trp D4	NBD D3	Trp D4	Oligo SDS
**cholesterol**	1.0	1.0	1.0	1.0	1.0	+++
**7-dehydrocholesterol**	(1.7)	0.67	quenched	0.89	quenched	
**dihydrocholesterol**	0.50	0.46	0.83		1.1	+++
**β-sitosterol**	0.50	0.61	0.59	0.80	0.94	+++
**lathosterol**	0.50				0.85	+++
**allocholesterol**		0.40			0.68	++
**desmosterol**		0.18			1.2	+++
**coprostanol**	0.71	0.11			0.65	+
**zymosterol**					0.61	+
**ergosterol**	0.10	0.13	quenched	0.44	quenched	
**fucosterol**			0.42			
**stigmasterol**	0.33	0.037	low	<0.08		

Numbers in the table show the relative effect when compared with the one observed for cholesterol. Hemolysis inhibition is calculated using the Inhibitory dose 50 (I_50_) reported for SLO [34] or PFO [36]. Values for the interaction of PFO with sterols in aqueous buffer were calculated using the concentration of sterol that cause half of the total change in Trp emission (nPFO, Figure 5 and Ref. [33]) or NBD emission (rPFO^V322C-NBD^, Figure 6 and Ref. [33]). Sterol in liposomes values were calculated using the mol % sterol that cause half of the total change in Trp emission for rPFO [9]. Relative values for rPFO oligomerization were estimated using the SDS-agarose gel electrophoresis analysis done by Nelson et al. [9].

## Supplementary Materials

The following are available online at www.mdpi.com/2072-6651/9/12/381/s1, Figure S1: Absorbance spectra of sterols in ethanol; Figure S2: Sequence and structure similarity between D4 domains of PFO and PLY.

## References

[1] Tweten R.K. (2005). Cholesterol-dependent cytolysins, a family of versatile pore-forming toxins. Infect. Immun..

[2] Heuck A.P., Moe P.C., Johnson B.B., Harris J.R. (2010). The cholesterol-dependent cytolysins family of Gram-positive bacterial toxins. Cholesterol Binding Proteins and Cholesterol Transport.

[3] Johnson B., Heuck A., Anderluh G., Gilbert R. (2014). Perfringolysin O structure and mechanism of pore formation as a paradigm for cholesterol-dependent cytolysins. Macpf/cdc Proteins—Agents of Defence, Attack and Invasion.

[4] Giddings K.S., Zhao J., Sims P.J., Tweten R.K. (2004). Human CD59 is a receptor for the cholesterol-dependent cytolysin intermedilysin. Nat. Struct. Mol. Biol..

[5] Giddings K.S., Johnson A.E., Tweten R.K. (2003). Redefining cholesterol’s role in the mechanism of the cholesterol-dependent cytolysins. Proc. Natl. Acad. Sci. USA.

[6] Alving C.R., Habig W.H., Urban K.A., Hardegree M.C. (1979). Cholesterol-dependent tetanolysin damage to liposomes. Biochim. Biophys. Acta.

[7] Rosenqvist E., Michaelsen T.E., Vistnes A.I. (1980). Effect of streptolysin O and digitonin on egg lecithin/cholesterol vesicles. Biochim. Biophys. Acta.

[8] Ohno-Iwashita Y., Iwamoto M., Ando S., Iwashita S. (1992). Effect of lipidic factors on membrane cholesterol topology—Mode of binding of θ-toxin to cholesterol in liposomes. Biochim. Biophys. Acta.

[9] Nelson L.D., Johnson A.E., London E. (2008). How interaction of perfringolysin O with membranes is controlled by sterol structure, lipid structure, and physiological low ph: Insights into the origin of perfringolysin O-lipid raft interaction. J. Biol. Chem..

[10] Flanagan J.J., Tweten R.K., Johnson A.E., Heuck A.P. (2009). Cholesterol exposure at the membrane surface is necessary and sufficient to trigger perfringolysin O binding. Biochemistry.

[11] Ramachandran R., Heuck A.P., Tweten R.K., Johnson A.E. (2002). Structural insights into the membrane-anchoring mechanism of a cholesterol-dependent cytolysin. Nat. Struct. Mol. Biol..

[12] Soltani C.E., Hotze E.M., Johnson A.E., Tweten R.K. (2007). Structural elements of the cholesterol-dependent cytolysins that are responsible for their cholesterol-sensitive membrane interactions. Proc. Natl. Acad. Sci. USA.

[13] Farrand A.J., LaChapelle S., Hotze E.M., Johnson A.E., Tweten R.K. (2010). Only two amino acids are essential for cytolytic toxin recognition of cholesterol at the membrane surface. Proc. Natl. Acad. Sci. USA.

[14] Polekhina G., Giddings K.S., Tweten R.K., Parker M.W. (2005). Insights into the action of the superfamily of cholesterol-dependent cytolysins from studies of intermedilysin. Proc. Natl. Acad. Sci. USA.

[15] Moe P.C., Heuck A.P. (2010). Phospholipid hydrolysis caused by *Clostridium perfringens* α-toxin facilitates the targeting of perfringolysin o to membrane bilayers. Biochemistry.

[16] Johnson B.B., Moe P.C., Wang D., Rossi K., Trigatti B.L., Heuck A.P. (2012). Modifications in perfringolysin O domain 4 alter the cholesterol concentration threshold required for binding. Biochemistry.

[17] Lally C.C.M., Bauer B., Selent J., Sommer M.E. (2017). C-edge loops of arrestin function as a membrane anchor. Nat. Commun..

[18] Saunders F.K., Mitchell T.J., Walker J.A., Andrew P.W., Boulnois G.J. (1989). Pneumolysin, the thiol-activated toxin of *Streptococcus pneumoniae*, does not require a thiol group for in vitro activity. Infect. Immun..

[19] Pinkney M., Beachey E., Kehoe M. (1989). The thiol-activated toxin streptolysin O does not require a thiol group for cytolytic activity. Infect. Immun..

[20] Michel E., Reich K.A., Favier R., Berche P., Cossart P. (1990). Attenuated mutants of the intracellular bacterium *Listeria monocytogenes* obtained by single amino acid substitutions in listeriolysin O. Mol. Microbiol..

[21] Sekino-Suzuki N., Nakamura M., Mitsui K.-I., Ohno-Iwashita Y. (1996). Contribution of individual tryptophan residues to the structure and activity of θ-toxin (perfringolysin O), a cholesterol-binding cytolysin. Eur. J. Biochem..

[22] Korchev Y.E., Bashford C.L., Pederzolli C., Pasternak C.A., Morgan P.J., Andrew P.W., Mitchell T.J. (1998). A conserved tryptophan in pneumolysin is a determinant of the characteristics of channels formed pneumolysin in cells and planar lipid bilayers. Biochem. J..

[23] Billington S.J., Songer J.G., Jost B.H. (2002). The variant undecapeptide sequence of the *Arcanobacterium pyogenes* haemolysin, pyolysin, is required for full cytolytic activity. Microbiology.

[24] Dowd K.J., Tweten R.K. (2012). The cholesterol-dependent cytolysin signature motif: A critical element in the allosteric pathway that couples membrane binding to pore assembly. PLoS Pathog..

[25] Johnson B.B., Breña M., Anguita J., Heuck A.P. (2017). Mechanistic insights into the cholesterol-dependent binding of perfringolysin O-based probes and cell membranes. Sci. Rep..

[26] Park S.A., Park Y.S., Bong S.M., Lee K.S. (2016). Structure-based functional studies for the cellular recognition and cytolytic mechanism of pneumolysin from *Streptococcus pneumoniae*. J. Struct. Biol..

[27] Heuck A.P., Hotze E.M., Tweten R.K., Johnson A.E. (2000). Mechanism of membrane insertion of a multimeric β-barrel protein: Perfringolysin O creates a pore using ordered and coupled conformational changes. Mol. Cell.

[28] Bavdek A., Gekara N.O., Priselac D., Gutierrez Aguirre I., Darji A., Chakraborty T., MacÌŒek P., Lakey J.H., Weiss S., Anderluh G. (2007). Sterol and pH interdependence in the binding, oligomerization, and pore formation of listeriolysin O. Biochemistry.

[29] Waheed A., Shimada Y., Heijnen H.F.G., Nakamura M., Inomata M., Hayashi M., Iwashita S., Slot J.W., Ohno-Iwashita Y. (2001). Selective binding of perfringolysin O derivative to cholesterol-rich membrane microdomains (rafts). Proc. Natl. Acad. Sci. USA.

[30] Ohno-Iwashita Y., Shimada Y., Waheed A., Hayashi M., Inomata M., Nakamura M., Maruya M., Iwashita M. (2004). Perfringolysin O, a cholesterol-binding cytolysin, as a probe for lipid rafts. Anaerobe.

[31] Sokolov A., Radhakrishnan A. (2010). Accessibility of cholesterol in endoplasmic reticulum membranes and activation of SREBP-2 switch abruptly at a common cholesterol threshold. J. Biol. Chem..

[32] Olsen B.N., Bielska A.A., Lee T., Daily M.D., Covey D.F., Schlesinger P.H., Baker N.A., Ory D.S. (2013). The structural basis of cholesterol accessibility in membranes. Biophys. J..

[33] Heuck A.P., Savva C.G., Holzenburg A., Johnson A.E. (2007). Conformational changes that effect oligomerization and initiate pore formation are triggered throughout perfringolysin O upon binding to cholesterol. J. Biol. Chem..

[34] Prigent D., Alouf J.E. (1976). Interaction of streptolysin o with sterols. Biochim. Biophys. Acta.

[35] Kenneth C., Watson K.C., Kerr E.J. (1974). Sterol structural requirements for inhibition of streptolysin O activity. Biochem. J..

[36] Hase J., Mitsui K., Shonaka E. (1976). *Clostridium perfringens* exotoxins. Iv. Inhibition of the theta-toxin induced hemolysis by steroids and related compounds. Jpn. J. Exp. Med..

[37] Alouf J.E. (1980). *Streptococcal* toxins (streptolysin O, streptolysin S, erythrogenic toxin). Pharmacol. Ther..

[38] Megha, Bakht O., London E. (2006). Cholesterol precursors stabilize ordinary and ceramide-rich ordered lipid domains (lipid rafts) to different degrees: Implications for the bloch hypothesis and sterol biosynthesis disorders. J. Biol. Chem..

[39] Haberland M.E., Reynolds J.A. (1973). Self-association of cholesterol in aqueous solution. Proc. Natl. Acad. Sci. USA.

[40] Gimpl G., Burger K., Fahrenholz F. (1997). Cholesterol as modulator of receptor function. Biochemistry.

[41] Nelson L.D., Chiantia S., London E. (2010). Perfringolysin O association with ordered lipid domains: Implications for transmembrane protein raft affinity. Biophys. J..

[42] Ramachandran R., Tweten R.K., Johnson A.E. (2004). Membrane-dependent conformational changes initiate cholesterol-dependent cytolysin oligomerization and intersubunit beta-strand alignment. Nat. Struct. Mol. Biol..

[43] Sherman W., Day T., Jacobson M.P., Friesner R.A., Farid R. (2006). Novel procedure for modeling ligand/receptor induced fit effects. J. Med. Chem..

[44] Feil S.C., Ascher D.B., Kuiper M.J., Tweten R.K., Parker M.W. (2014). Structural studies of *Streptococcus pyogenes* streptolysin O provide insights into the early steps of membrane penetration. J. Mol. Biol..

[45] Gilbert R., Anderluh G., Gilbert R. (2014). Structural features of cholesterol dependent cytolysins and comparison to other MACPF-domain containing proteins. MACPF/CDC Proteins—Agents of Defence, Attack and Invasion.

[46] Chou P.Y., Fasman G.D. (1978). Empirical predictions of protein conformation. Ann. Rev. Biochem..

[47] Kulma M., Kacprzyk-Stokowiec A., Kwiatkowska K., Traczyk G., Sobota A., Dadlez M. (2017). R468A mutation in perfringolysin O destabilizes toxin structure and induces membrane fusion. Biochim. Biophys. Acta (BBA) Biomembr..

[48] Shelley J.C., Cholleti A., Frye L.L., Greenwood J.R., Timlin M.R., Uchimaya M. (2007). Epik: A software program for pKa prediction and protonation state generation for drug-like molecules. J. Comput.-Aided Mol. Des..

[49] Farrand A.J., Hotze E.M., Sato T.K., Wade K.R., Wimley W.C., Johnson A.E., Tweten R.K. (2015). The cholesterol-dependent cytolysin membrane-binding interface discriminates lipid environments of cholesterol to support β-barrel pore insertion. J. Biol. Chem..

[50] Cho W., Stahelin R.V. (2005). Membrane-protein interactions in cell signaling and membrane trafficking. Annu. Rev. Biophys. Biomol. Struct..

[51] Xu S., Benoff B., Liou H.-L., Lobel P., Stock A.M. (2007). Structural basis of sterol binding by NPC2, a lysosomal protein deficient in Niemann-Pick typeC2 disease. J. Biol. Chem..

[52] Rezai T., Bock J.E., Zhou M.V., Kalyanaraman C., Lokey R.S., Jacobson M.P. (2006). Conformational flexibility, internal hydrogen bonding, and passive membrane permeability: Successful in silico prediction of the relative permeabilities of cyclic peptides. J. Am. Chem. Soc..

[53] Shepard L.A., Heuck A.P., Hamman B.D., Rossjohn J., Parker M.W., Ryan K.R., Johnson A.E., Tweten R.K. (1998). Identification of a membrane-spanning domain of the thiol-activated pore-forming toxin *Clostridium perfringens* perfringolysin O: An α-helical to β-sheet transition identified by fluorescence spectroscopy. Biochemistry.

