# Supplementary material for: Interaction of Cholesterol with Perfringolysin O: What Have We Learned from Functional Analysis?
Source: Toxins (Basel). 2017 Nov 23;9(12):381. doi: 10.3390/toxins9120381 (PMC5744101; doi:10.3390/toxins9120381)
Supplement: Supplementary file 1 [file toxins-09-00381-s001.pdf]

Supplementary Materials: Interaction of Cholesterol with Perfringolysin O: What Have We Learned from Functional Analysis?

Sergey N. Savinov and Alejandro P. Heuck

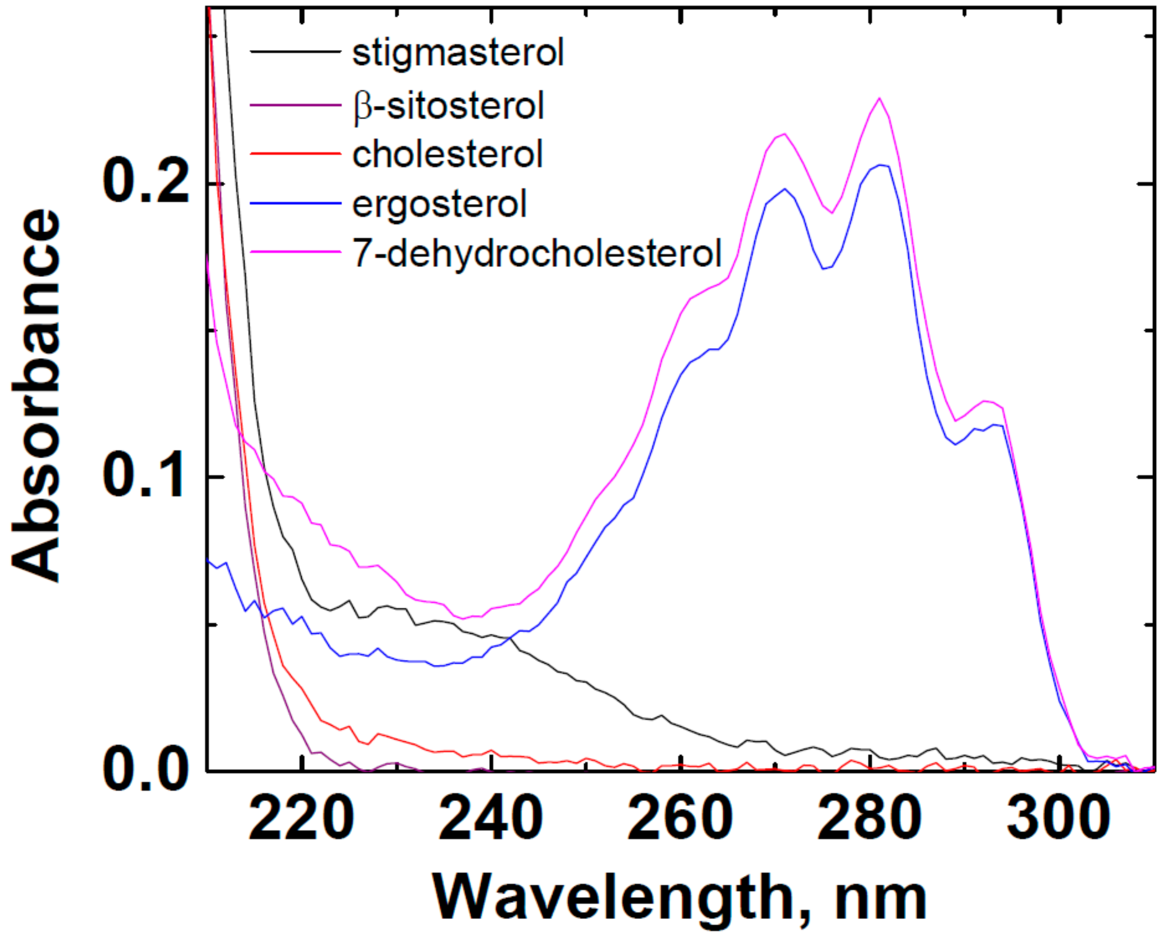

Figure S1. Absorption spectra for the indicated sterols in ethanol. The concentration was stigmasterol 2 mM, β-sitosterol 0.2 mM, cholesterol 0.15 mM, 7-dehydrocholesterol and ergosterol 0.02 mM. Light path 1 cm.

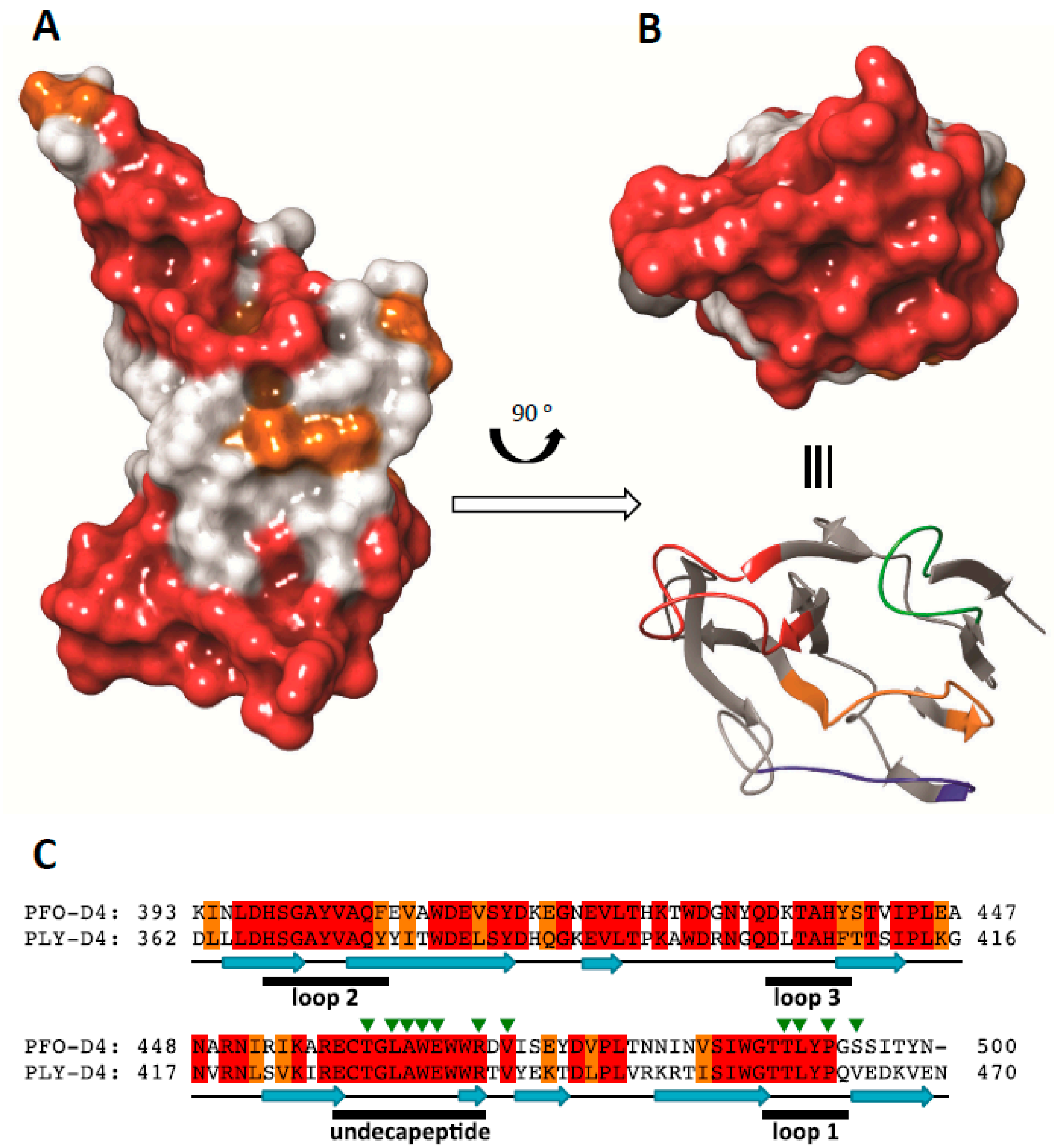

Figure S2. Sequence and structure similarity between D4 domains of PFO and PLY. Panel A shows a side view of PFO D4 (PDB ID: 1PFO) rendered as molecular surface and colored by conservation between PFO and PLY (red: identical, orange: similar, white: non-conserved). Panel B shows the perpendicular view produced by 90° rotation around the horizontal axis within the figure plane and illustrates the bottom of the distal end of the CDCs. The top and bottom images are rendered as a molecular surface and cartoon ribbon, respectively. The membrane-binding loops are indicated. The surface colors are based on sequence alignment (panel C) for PFO and PLY D4 with the same color labeling of residues. The loops in the cartoon ribbon image are color coded as in Figure 1. Panel C shows the alignment of the amino acid sequences for PFO and PLY D4, with secondary structure (blue arrows), loop positions, and proximal (within 4 Å) residues for cholesterol–PLY-D4 binding model (green triangles) indicated.
